# Response to Letter to the Editor

**DOI:** 10.1016/j.ejvsvf.2022.10.003

**Published:** 2022-10-22

**Authors:** Elise Beijer, Arjan W.J. Hoksbergen

**Affiliations:** Department of Vascular Surgery, Amsterdam University Medical Centres, Amsterdam, the Netherlands

We would like to thank S Duvnjak et al. for their comments. In our case report,^1^ we elaborated on a complicated case in which the patient developed vascular graft infection years after endovascular repair of an acutely bleeding aortic stump. We stated that in such cases the endovascular salvage repair should be followed by a more definitive treatment when the patient is fit for surgery, especially for of aortoduodenal fistula cases. However, we also expressed our thoughts on patients who are unfit for surgery; they may well benefit from a more conservative approach with culture based antibiotic therapy, making this second best treatment option a reasonable alternative.

S Duvnjak et al. described a case^2^ in which their patient died two years after endovascular intervention for an aortic stump bleed due to a new bleeding complication. This case adds to our above mentioned perspective and suggests that personalised medicine plays a crucial role in the treatment of these vulnerable patients.

## References

[1] Beijer E., Scholtes V.P.W., Moerbeek P., Coveliers H.M.E., Lely R.J., Hoksbergen A.W.J. (2020 Apr 13). Endovascular treatment of aortic stump blow-out after extra-anatomical repair of aortoduodenal fistula: a case report and review of literature. CVIR Endovasc.

[2] Duvnjak S., Andersen P.E., Larsen K.E., Roeder O. (2014 Aug). Endovascular repair of postoperative vascular graft related complications after aorto-iliac surgery. Int Angiol.

